# The Effect of Cadmium on the Activity of Stress-Related Enzymes and the Ultrastructure of Pea Roots

**DOI:** 10.3390/plants8100413

**Published:** 2019-10-14

**Authors:** Katarzyna Głowacka, Anna Źróbek-Sokolnik, Adam Okorski, Janusz Najdzion

**Affiliations:** 1 Department of Plant Physiology, Genetics and Biotechnology, Faculty of Biology and Biotechnology, University of Warmia and Mazury in Olsztyn, 10-719 Olsztyn, Poland; 2 Department of Botany and Nature Protection, Faculty of Biology and Biotechnology, University of Warmia and Mazury in Olsztyn, 10-727 Olsztyn, Poland; a.zrobeksokolnik@uwm.edu.pl; 3 Department of Entomology, Phytopathology and Molecular Diagnostics, University of Warmia and Mazury in Olsztyn, 10-727 Olsztyn, Poland; adam.okorski@uwm.edu.pl; 4 Department of Animal Anatomy and Physiology, Faculty of Biology and Biotechnology, University of Warmia and Mazury in Olsztyn, 10-727 Olsztyn, Poland; jnajdzion@uwm.edu.pl

**Keywords:** antioxidative enzyme, cadmium, phenylalanine ammonia-lyase, plastolysome, starch, suberin

## Abstract

The analysis of the effects of cadmium (Cd) on plant cells is crucial to understand defense mechanisms and adaptation strategies of plants against Cd toxicity. In this study, we examined stress-related enzyme activities after one and seven days of Cd application and the ultrastructure of roots of *Pisum sativum* L. after seven days of Cd treatment (10, 50, 100, and 200 μM CdSO_4_). Our results showed that phenylalanine ammonia-lyase (PAL) activity and the amount of Cd accumulated in the roots were significantly positively correlated with the Cd concentration used in our experiment. However, Cd caused a decrease of all studied antioxidative enzyme activities (i.e., catalase (CAT), ascorbate peroxidase (APX), guaiacol peroxidase (GPX)). The analysis of the ultrastructure (TEM) showed various responses to Cd, depending on Cd concentrations. In general, lower Cd concentrations (50 and 100 μM CdSO_4_) mostly resulted in increased amounts of oil bodies, plastolysomes and the accumulation of starch granules in plastids. Meanwhile, roots treated with a higher concentration of Cd (200 μM CdSO_4_) additionally triggered protective responses such as an increased deposition of suberin lamellae in the endodermal cell walls. This indicates that Cd induces a complex defense response in root tissues.

## 1. Introduction

Cadmium (Cd) is a widespread heavy metal, released into the environment mostly by anthropogenic pressure (power stations, heating systems, metal-working industries, waste incinerators, urban traffic, cement factories, and as a by-product of phosphate fertilizers) but also as a result of the rock mineralization processes [1,2]. The toxic effects of Cd on plants have been studied and described in many review papers [3,4,5,6,7]. Universal or common features involved in plant responses to the Cd application are increased production of toxic oxygen derivatives and alterations of the activity of the antioxidant defense system, which must be proportional to the task of the destruction of reactive oxygen species (ROS) during normal metabolism and during stress [8,9,10,11]. The plant antioxidant defense system includes a variety of antioxidant molecules and enzymes. Changes in the activity of this system can give an inside view of the quantity of stress that plants are under [12]. It was shown that the application of 50 µM Cd decreases catalase (CAT), glutathione reductase, and guaiacol peroxidase (GPX) activities but increases superoxide dismutase activity after 14 days of Cd treatment in pea roots [13]. Moreover, low doses of Cd (5 µM) increased CAT and peroxidase activities, while ascorbate peroxidase activity (APX) in some pea genotypes was increased, reduced, or not affected after 10 days of Cd treatment [14]. It should be noted though, that the effects of short-term Cd exposure on pea roots were not studied in these experiments. Additionally, the localization of ROS and vascular tissue in the roots of *Pisum sativum* supplemented with 50 µM Cd, showed differences between Cd-treated and control root, suggesting cell wall peroxidases as important sources of ROS in the process of lignification [13]. The Cd-induced increase of lignin content was in fact correlated with phenylalanine ammonia-lyase (PAL) activity in soybean roots [15]. However, the decreased lignin accumulation in lupine roots in response to heavy metals, despite an increased PAL activity, suggests that the activated phenylpropanoid pathway was involved in the synthesis of secondary metabolites other than lignin [15]. Significant increase in total phenolic content and PAL activity was shown during Cd treatment in fronds of *Azolla imbricata* [16]. Furthermore, the PAL coding gene was detected among Cd-induced genes in the heavy metal accumulator *Brassica juncea* [17]. In contrast, the expression of PAL did not change significantly in pea leaves after 50 µM Cd treatment [18]. It is important to note, however, that pea roots were not examined in these experiments. Higher plants can uptake Cd directly from the soil [19]. Therefore, the roots are the precise location of primary contact where Cd is absorbed [20]. Pea plants grown with 50 µM CdCl_2_ accumulated Cd mainly in the roots, whose growth was 1.36-fold reduced [21]. It was shown that anatomical alterations, which can be a result of Cd treatment, can change accumulation processes and vegetative growth of the plants when they are exposed to this metal [22,23]. Development of Casparian strips and suberin lamellae was observed in the roots of different *Salix* clones with various properties of Cd accumulation and Cd tolerance [24]. Similar results were also observed in the roots of maize [20] and *Tritonia gladiolaris* [23]. Additionally, it was shown that Cd induces the formation of a hypodermal periderm in the roots of the monocotyledonous medicinal plant *Merwilla plumbea* [22]. It is important to notice that involvement of the suberization process of the cell walls of Cd-treated pea roots has not been determined. Moreover, little is known about the ultrastructure of root cells exposed to Cd. There are only a few studies reporting the effect of Cd on the root plastids [25] or on the ultrastructure of the root meristematic cells [26,27], whereas most studies describe the alterations of the ultrastructure of leaves caused by Cd application [21,26,27,28,29,30,31]. It should also be stressed that most literature on this topic focuses on seedlings, mostly 7 [14,16] or 14 days old [13,21], while our study was to analyze older plants (four weeks old). The knowledge of how mature plants cope with Cd treatment seems to be very important, particularly in a situation of progressive environmental pollution.

Therefore, the aim of this study was to determine the broad effects of Cd treatment (with 10, 50, 100, and 200 µM CdSO_4_) on four-week-old pea roots. In order to achieve this goal, we analyzed the ultrastructure of lateral roots after seven days of Cd application. We also examined the activity of PAL and three antioxidative enzymes i.e., CAT, APX, and GPX after one and seven days of Cd treatment. Moreover, the localization of H_2_O_2_ was also examined after Cd application. We believe that these results could be helpful for better understanding the cytotoxicity of Cd on plant tissues. Furthermore, since *P. sativum* L. is an important plant in agriculture and the food industry, and it is widely cultivated in many countries, the present results could be of practical value for scientists in the field of experimental botany, plant physiology, and plant–soil interactions.

## 2. Results

### 2.1. Accumulation of Cd in Pea Roots

**Figure 1:**
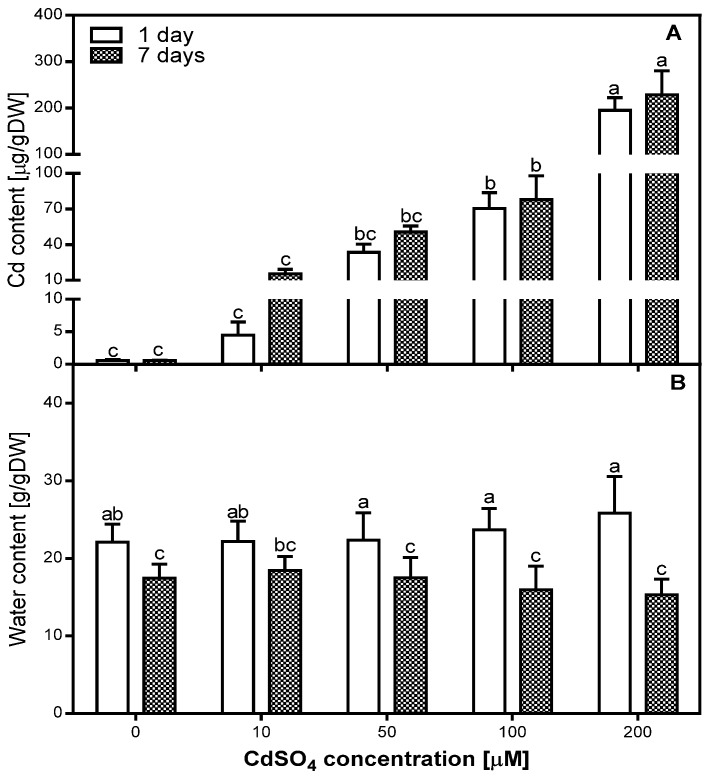
Accumulation of Cd (**A**) and water content (**B**) in the control (0) and Cd-treated (10, 50, 100, and 200 µM CdSO_4_) roots of *Pisum sativum*, after one and seven days of Cd treatment. Each value is the mean of three replicates ± SD. Different letters represent significant differences (*p* < 0.01).

The analysis of Cd content showed a gradual accumulation of this metal in pea roots (Figure 1A). The amount of Cd in the root tissues was significantly positively correlated with the concentration of Cd applied during the experiment after both one (R = 0.98) and seven days (R = 0.95). However, the absorption rate of low doses of Cd was higher than high doses. In the roots of 10 and 50 µM Cd-treated plants, after seven days of treatment, the amount of Cd was higher by 245% and 51%, respectively, compared to one day of Cd treatment. In the case of 100 and 200 µM Cd-treated roots, the amount of Cd measured after one day was close to the maximum, since it increased after seven days only by 11% and 17%, respectively.

### 2.2. Effects of Cd on the Water Content in Pea Roots

The analysis of water content (WC) in the roots of *P. sativum* after one and seven days of Cd treatment did not show significant differences between control and Cd-treated roots (Figure 1B). We determined that WC in all variants of the experiment was lower after seven days than after one day of Cd treatment. This decrease was greater in 100 and 200 µM Cd-treated roots (lower by 32% and 40%, respectively) than in the control, as well as in 10 and 50 µM Cd-treated roots (lower by 21%, 17%, and 21%, respectively). Statistical analysis of correlation between WC and Cd concentration showed a low positive correlation (R = 0.4203) after one day and a low negative correlation (R = −0.438) after seven days of Cd treatment. 

### 2.3. Effects of Cd on PAL Activity in Pea Roots

**Figure 2:**
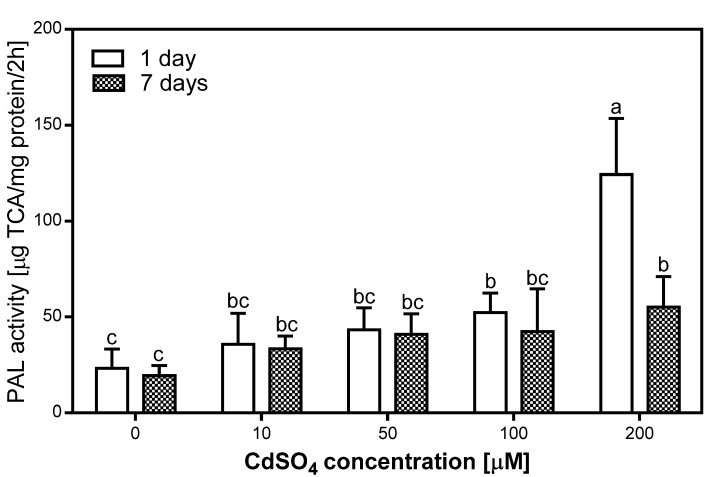
Phenylalanine ammonia-lyase (PAL) activity in the control (0) and Cd-treated (10, 50, 100, and 200 µM CdSO_4_) roots of *P. sativum*, after one and seven days of Cd treatment. Each value is the mean of three replicates ± SD. Different letters represent significant differences (*p* < 0.01).

The activity of PAL was measured in the roots of pea plants after one and seven days of Cd treatment (Figure 2). We evaluated that PAL activity increased in the tissues of Cd-treated plants (Figure 2). The highest level of PAL activity was noticed after one day of 200 µM Cd treatment, and it was five times higher than in the control. In comparison, PAL activity measured in the 100 µM Cd-treated roots was 2.5 times higher than in the control roots. Figure 2 shows that PAL activity decreased after one week of treatment, the most in the 200 µM Cd-treated roots, where it was 60% lower than after one day, but still three times higher than in the control roots. With regards to the 100 µM Cd-treated roots, after seven days of Cd treatment, the PAL activity was 20% lower than after one day. Interestingly, the analyzed enzyme activity in the 10 and 50 µM Cd-treated roots was relatively stable and it was 1.5–2 times higher than in the control roots. The statistical analysis also showed that the level of PAL activity increased after one day of Cd treatment with a correlation coefficient of R = 0.89. Meanwhile, after seven days of Cd treatment the correlation coefficient was R = 0.84.

### 2.4. Effects of Cd on Antioxidative Enzyme Activity in Pea Roots

**Figure 3:**
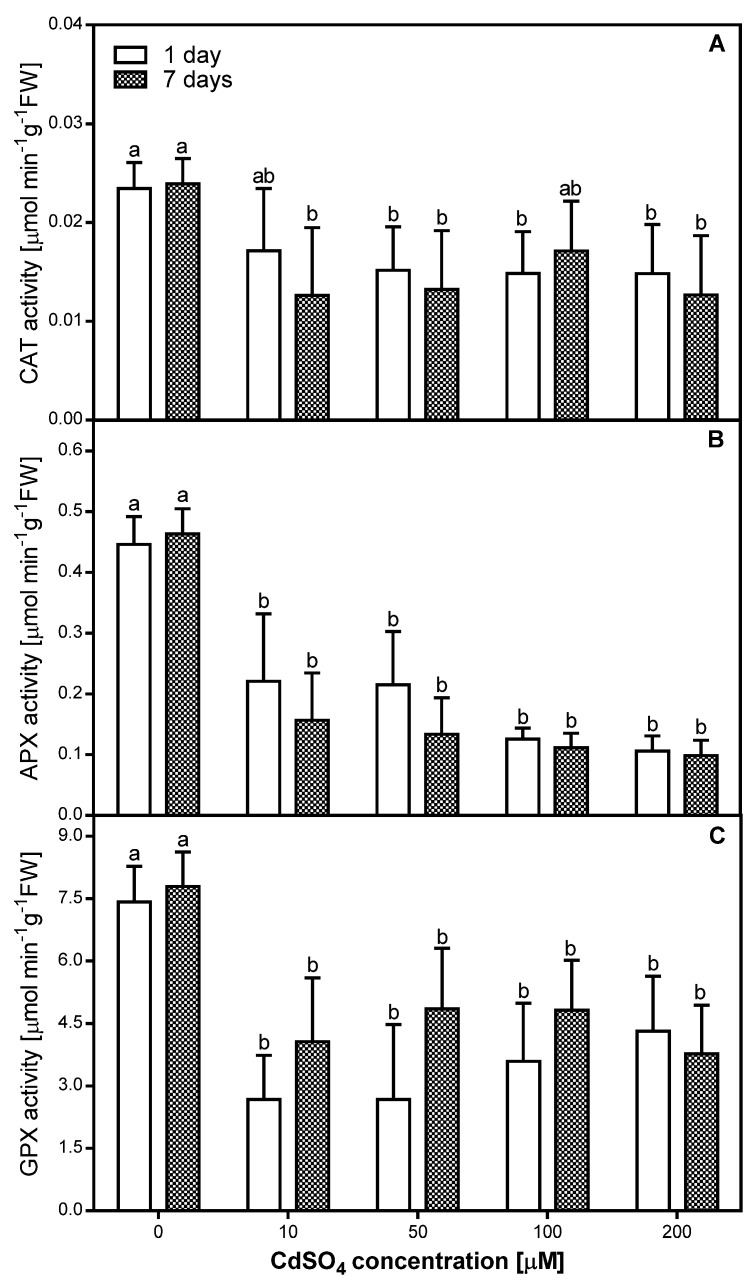
Catalase (CAT) (**A**), ascorbate peroxidase (APX) (**B**), and guaiacol peroxidase (GPX) (**C**) activity in the control (0) and Cd-treated (10, 50, 100, and 200 µM CdSO_4_) roots of *P. sativum*, after one and seven days of Cd treatment. Each value is the mean of three replicates ± SD. Different letters represent significant differences (*p* < 0.01).

Changes induced by short- and long-term exposure to Cd in different antioxidative enzymes involved in cellular defense were studied at the level of enzyme activity. Overall, the activity of all antioxidative enzymes decreased in the roots of *P. sativum* after one and seven days of Cd treatment (Figure 3).

The highest CAT activity was observed in samples exposed to 10 µM CdSO_4_ in the course of a one-day treatment and to 100 µM in the course of seven-days treatment—the equivalent diminutions from controls were approximately 30% in both cases (Figure 3A). In the other cases, the CAT activity level was almost constant and averaged approximately 60% of the control level when plants were treated with Cd concentrations from 50 to 200 µM for one day or approximately 53% of control level when plants were treated with 10, 50, or 200 µM Cd for seven days (Figure 3A). A significant correlation (R = −0.64) with Cd concentration used in the experiment was observed only after one day of Cd application.

The presented data indicate that the highest and simultaneously comparable APX activity level (Figure 3B) was observed in pea roots treated with 10 and 50 µM of CdSO_4_ for one day (approximately 50% of the control level). Exposure to 100 or 200 µM CdSO_4_ for one day as well as to 10–200 µM CdSO_4_ for seven days resulted in the strongest inhibitory modification in the APX activity within pea roots—equivalent diminutions from controls were approximately 70%. It is also important to note that only in the case of APX activity, a significant negative correlation was observed after both one (R = −0.75) and seven (R = −0.61) days of Cd treatment. 

In the case of GPX, a lower activity was observed after one day of Cd treatment than after seven days of Cd treatment, with the exception of 200 µM CdSO_4_ (Figure 3C). In the roots of pea exposed to Cd for one day, we observed a slight and progressive increase in GPX activity along with an increase of metal concentration used. The activity of GPX in roots of pea exposed to Cd for seven days was the highest and comparable when 50 and 100 µM CdSO_4_ was tested (approximately 62% of control level), and slightly lower when 10 or 200 µM CdSO_4_ was tested (approximately 50% of control level). A significant negative correlation was observed only after seven days of Cd treatment (R = −0.58).

### 2.5. Effects of Cd on H_2_O_2_ Localization in Pea Roots

**Figure 4:**
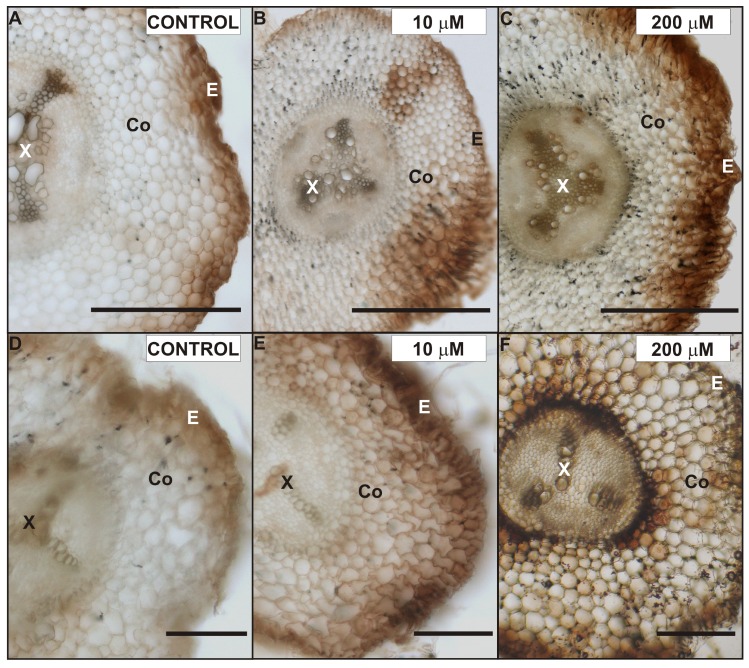
Localization of H_2_O_2_ in the primary (**A**–**C**) and lateral roots (**D**–**F**) of control (**A**,**D**), 10 µM (**B**,**E**) and 200 µM (**C**,**F**) CdSO_4_-treated *P. sativum* after seven days of treatment. Abbreviations: E—epidermis, Co—cortex, X—xylem. Scale bar: 100 µm.

The hydrogen peroxide (H_2_O_2_) localization was analyzed in the roots of control and Cd-treated pea plants after seven days of Cd application with a modified DAB-dependent method (Figure 4). A characteristic reddish-brown coloration, indicating H_2_O_2_ occurrence, was localized in root tissues in the primary (Figure 4A–C) as well as the lateral (Figure 4D–F) roots. In the control, H_2_O_2_ was observed mostly in the xylem cell walls, cortex external cells, and epidermis (Figure 4A,D), while in the roots exposed to Cd a strong H_2_O_2_–DAB dependent reaction was also seen in the entire cortex, especially in cells located closer to the stele (Figure 4B,C,E,F). Moreover, the H_2_O_2_ coloration in the cells and cell walls of the Cd-treated roots was noticeably stronger than in that of the control roots. It is also important to note, that the highest level of H_2_O_2_ was seen in the cortex and endodermis cell walls of the lateral roots exposed to 200 µM CdSO_4_ (Figure 4F).

### 2.6. Effects of Cd on the Lateral Root Ultrastructure

**Figure 5:**
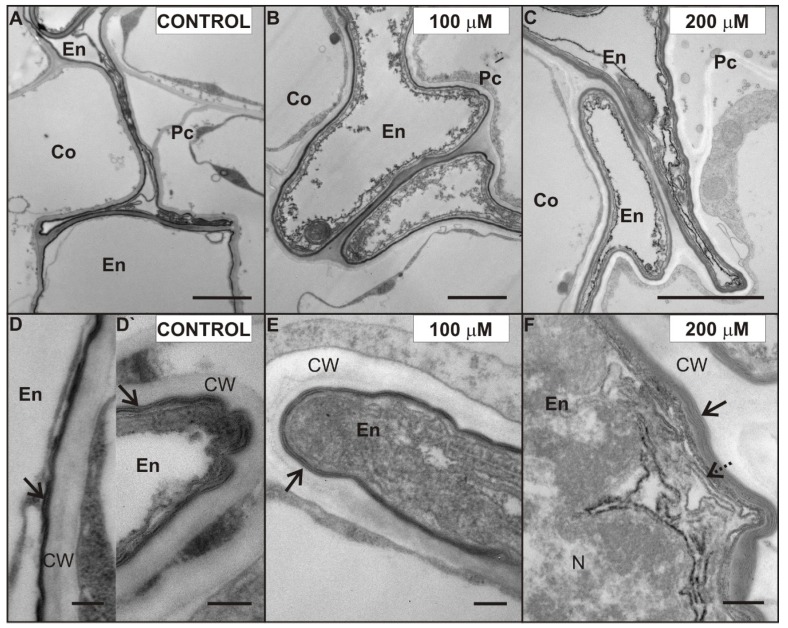
Ultrastructure of suberized endodermal cells in the control (**A**), 100 (**B**), and 200 (**C**) µM CdSO_4_-treated roots of *P. sativum* after seven days of Cd treatment. The fine structure of electron-translucent suberin lamellae (arrows) of the endodermal cells in the control (**D**,**D`**), 100 (**E**), and 200 (**F**) µM Cd-treated roots. New suberin lamellae (dotted arrow). Abbreviations: Co—cortex, En—endodermis, Pc—pericycle, CW—cell wall, N—nucleus. Scale bar **A**–**C**: 2 µm, **D**–**F**: 0.2 µm.

**Figure 6:**
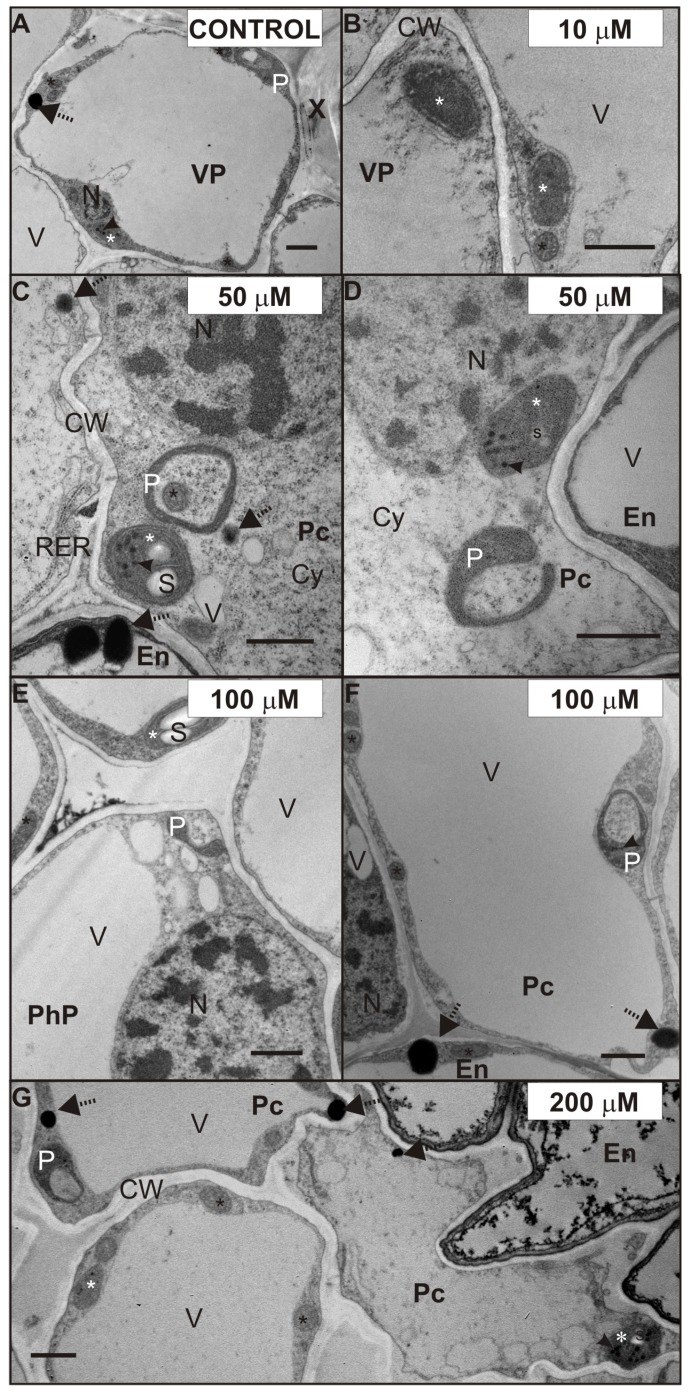
Ultrastructure of plastids of the control (**A**), 10 (**B**), 50 (**C**,**D**), 100 (**E**,**F**), and 200 (**G**) µM CdSO_4_-treated root stele of *P. sativum* after seven days of Cd treatment. Plastids (white stars), plastoglobuli (arrowheads), mitochondria (black stars), and oil bodies (dotted arrows). Abbreviations: Co—cortex; CW—cell walls; Cy—cytosol; IS—intercellular space, En—endodermis; N—nucleus; P—plastolysome; Pc—pericycle; PhP—phloem parenchyma; RER—rough endoplasmic reticulum; s—starch granule; V—vacuole; VP—vascular parenchyma; X—xylem. Scale bar: 1 µm.

**Figure 7:**
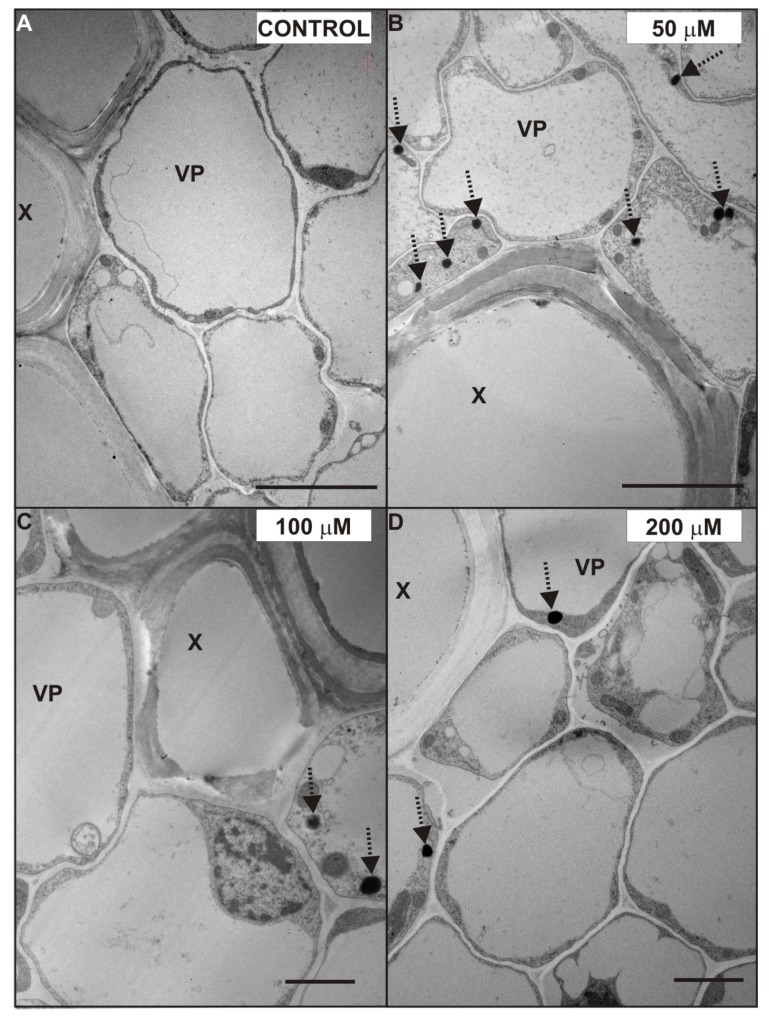
Ultrastructure of xylem vessels and vascular parenchyma cells of the control (**A**) and 50 (**B**), 100 (**C**), and 200 (**D**) µM CdSO_4_-treated roots of *P. sativum* after seven days of Cd treatment. Oil bodies (dotted arrows). Abbreviations: X—xylem, VP—vascular parenchyma. Scale bar **A**, **B**: 5 µm; **C**, **D**: 2 µm.

We decided to investigate the possible ultrastructure alterations caused by Cd in the lateral roots (Figure 5, Figure 6 and Figure 7). The reason for that was the fact that the lateral roots treated with Cd displayed a higher level of H_2_O_2_, which suggested intense cellular reactions elicited by Cd application (Figure 4). Furthermore, the lateral roots formed a major portion of a complex root system of four-week-old pea plants used in the experiment. The results of ultrastructure observation revealed that Cd had induced changes in the cells of the endodermis (Figure 5) and stele (Figure 6 and Figure 7).

One of the characteristic effects of Cd application was alterations in the ultrastructure of endodermal cells, possibly induced by Cd (Figure 5). The endodermis was formed by a single cell layer. Some cells in this layer were misshapen and they showed electron dense cytoplasm (Figure 5). Moreover, cell walls of these misshapen cells were suberized, but a different number of suberin lamellae was observed. Generally, we noticed two or three suberin lamellae (arrow) encrusting the endodermal cell walls of the control (Figure 5D) and 100 µM Cd-treated roots (Figure 5E), while even up to six suberin lamellae were seen in the cell walls of the 200 µM Cd-treated pea roots (Figure 5F). What is more, we noticed a formation of new lamellae in the cytoplasm also (Figure 5F, dotted arrow) of root cells treated with 200 µM Cd. Interestingly, in the suberized cell walls of the uncollapsed endodermal cells of control roots, only one, not fully developed, suberin lamella was observed (Figure 5D`).

The ultrastructure of root stele cells also showed some interesting features, which were possibly caused by exposure to Cd. Figure 6 shows the ultrastructure of plastids (white star), after seven days of Cd treatment. Shapes of plastids observed in the control (Figure 6A) and Cd-treated roots (Figure 6B–E,G) were mostly ellipsoidal with plastoglobuli (arrowhead). However, often the plastids in pericycle cells (Figure 6C,D,F,G), phloem parenchyma (Figure 6E, PhP) or VP cells (Figure 6A,E) formed pockets enclosing the mitochondria (Figure 6C, black star) or cytoplasm (Figure 6D–G). We observed these plastids in the Cd-treated roots frequently and considered them as plastolysomes (P). It is worth mentioning, that plastids forming similar structures were also noticed in VP cells in the control roots (Figure 6A). Furthermore, it should be noticed that in the case of 100 and 200 µM Cd-treated roots, the stroma of plastolysomes was denser (Figure 6F,G). Moreover, it was characteristic that in the stele cells of the 50–200 µM (Figure 6C–E,G) and cortex cells of 200 µM Cd-treated roots, some of the plastids contained starch (s) granules.

Another effect of Cd treatment, noticed in almost all tissues of the root, was an increased number of oil bodies (Figure 6 and Figure 7, dotted arrows). The oil bodies were mainly observed in the cytosol of the VP cells treated with 50 (Figure 7B), 100 (Figure 7C), and 200 (Figure 7D) µM CdSO_4_. Additionally, these lipid-containing structures were also noticed in the cytoplasm of other stele cells (Figure 6C,F,G), and in the cortex cells of 200 µM Cd-treated roots. However, it should be mentioned that oil bodies were observed in the VP cells of the control root (Figure 6A), but their number was smaller than in those treated with Cd.

## 3. Discussion

The present results show that Cd treatment of four-week-old pea plants changes the activity of stress-related enzymes and the ultrastructure of root cells. Moreover, the effects we observed are complex and depend on the concentration of Cd used in the experiment. First of all, our data show that PAL activity was increased after Cd treatment. However, it was specifically observed in the case of the 200 µM Cd-stressed root, after one day of Cd application, and it was the highest from all analyzed variants of the experiment. Increased PAL activity was reported also in the roots of four-day-old soybean (*Glycine max* L.) and lupine (*Lupinus luteus* L.) seedlings treated with Cd for 48 h [15]. In contrast with the effect on PAL activity, all concentrations of Cd used in our experiment caused the decrease of CAT, AXP, and GPX activity. Moreover, the amount of H_2_O_2_ detected in the tissues of the primary and lateral roots was increased. The induction of activity in a particular group of enzymes is considered to play an important role in the cellular defense strategy against oxidative stress caused by toxic metal concentrations [32]. However, similar to our results, many studies have shown that Cd stress can lead to decreasing antioxidant capacity [21,33]. Moreover, under conditions where CAT and peroxidases are diminished, the cell is not fully competent to remove H_2_O_2_ which would accumulate in the tissues [13,33,34,35]. The accumulation of H_2_O_2_, also observed in our study, seems to be a crucial event during Cd treatment. Moreover, a Cd-dependent increase of APX activity was described in the roots of 15-day-old *P. sativum* by Dixit et al. [35]; while the opposite effect, which is similar to our results, was observed in five-week-old Scots pine roots by Schützendübel et al. [33]. Furthermore, similar to our results, a decrease of GPX activity was observed in pea roots by Rodríguez-Serrano et al. [13] and in pea leaves by Sandalio et al. [21]. Literature data relating to the impact of heavy metals on CAT are also heterogeneous. A reduction in CAT activity caused by the presence of Cd in pea plants was shown, for example, by Dixit et al. [35], Rodríguez-Serrano et al. [13], and Romero-Puertas et al. [36]. The opposite effect was observed in pea roots by Metwally et al. [14] and in the leaves of chickpea seedlings by Ahmad et al. [37]. It is important to note, that all these results were obtained in younger plants, which were treated with Cd longer than in our experiments. Furthermore, in contrast to our study, most of the above experiments [13,21,33,34,35,36] were conducted on plants cultivated in a greenhouse in aerated full-nutrient media under optimum conditions. We presume that cultivation conditions such as day length, frequency of watering, and nutrient composition could have an impact on the results of enzymes activity. Besides the cultivation conditions, there are other factors, that should not be overlooked. For example, our results show that those differences may also be caused by the stage of plant development. It should be pointed out that in our study we used mature plants, which may cope better with stresses and the magnitude of responses may differ. Overall, our results and data from the literature show that the direction of stress responses are not always identical, because they are vastly dependent on the plant species, the examined tissue, and the stress intensity.

The present results clearly show that Cd treatment of four-week-old pea plants changes the ultrastructure of root cells. We found that these changes differentiate according to the adequate concentration of heavy metal used in the experiment. We also noticed that the lowest concentration of Cd (10 µM CdSO_4_) caused the least damage to the root tissues. Consequently, significant ultrastructural alterations were observed in the cells of 50, 100, and 200 µM Cd-treated plants. We determined that the endodermal cell layer between cortex cells and the stele in the 200 µM Cd-stressed roots consisted of cells, whose cell walls showed more suberin lamellae than the control roots and the roots treated with smaller doses of Cd. Suberin enhancement of the cell walls of the roots exposed to Cd treatment was observed in many plants, namely maize [20], *T. gladiolaris* [23], the monocotyledonous medicinal plant *M. plumbea* [22] and different *Salix* clones with various properties of Cd accumulation and Cd tolerance [24]. It is known that suberization of the cell walls can affect solute transport to the stele and to the shoot [38]. Lux et al. [20] suggested that the accelerated maturation of the endodermis (Casparian band formation, suberization) was a Cd-induced process, which could protect the shoot from excessive Cd loads by reducing the entry of Cd to the xylem. Our results, showing suberization of the lateral roots and increasing accumulation of Cd after one and seven days of Cd treatment, support the theory of Cd-induced suberin deposition in the endodermal cell wall of *P. sativum* roots. Furthermore, we observed a higher gradual increase in the concentration of Cd in the roots treated with 10 and 50 µM CdSO_4_. In contrast, in plants treated with 100 and 200 µM CdSO_4_, the amount of Cd in the root tissue almost reached its maximum after one day of treatment and stayed on a similar level till the end of the experiment. These results suggest two things: First, that pea roots have a high capacity of Cd accumulation, and second that the absorption rate of the high doses of Cd decreased with exposure time. Hernández and Cooke [39] in their work showed that the concentration of Cd in pea plants treated with 50 µM reached 70% after one day, which corresponds with our results. They also indicated that a saturation phase was reached after three days. Our results demonstrate that the saturation phase of 100 and 200 µM Cd-treated plants was reached earlier, suggesting the induction of mechanisms, which can limit or impede Cd absorption. Moreover, it was described [40], that a fully suberized cell typically collapses during the dehydration and embedding procedures necessary for TEM, because of the low permeability of the suberized cell walls. We observed uncollapsed endodermal cells with suberized cell walls mostly in the control roots, where they showed usually only one not fully developed suberin lamellae. We presumed that a change in permeability of the cell walls may also affect the tissue water content but the differences in the level of water content between the control and Cd-treated plants were not significant. However, the decrease in the water content after seven days of Cd treatment was two times greater in 200 µM Cd-treated roots than in control roots. The Cd treatment increased the suberization of endodermal cell walls, and this can be also supported by our results regarding H_2_O_2_ localization and PAL activity. We observed an increased amount of H_2_O_2_ in the region of the root endodermis treated with Cd—especially with 200 µM CdSO_4_. Specifically, these cells had a greater number of suberin lamellae deposited in their walls. Similarly, Razem and Bernards [41] showed that H_2_O_2_ is required during wound-induced suberization of potato tubers. We also suggest, that the enhanced suberization of Cd-treated endodermal cell walls in the roots may be linked with PAL activity, since the aromatic moiety of suberin is synthesized via the general phenylpropanoid pathway with its key enzyme PAL [42].

The next interesting observation concerned the ultrastructure of plastids in the tissues of the Cd-stressed plant roots. We noticed that some of the plastids in the stele of 50, 100, and 200 µM Cd-treated roots elongated and sometimes surrounded mitochondria or cytoplasm. Considering our results and those reported by others, it seems that plastids observed in the cytoplasm of the pea root stele are plastolysomes. Autophagic plastids (plastolysomes) were first described by Nagl [43] in embryo suspensor in *Phaseolus*. The plastolysomes that we observed could be involved in the degradation of cytoplasm during cell differentiation but also could be stress related. Processes similar to those characteristic of macroautophagy, carried out by plastids, were also suggested by van Doorn et al. [44] and Parra-Vega et al. [45]. Filonova et al. [46] imply that plastolysome-like structures are precursors of autolytic vacuoles and consist of a portion of cytoplasm surrounded by one or several double membranes, with the sequestered cytoplasm characterized by increased electron translucency. In our study, this increased electron translucency of plastolysomes was observed in the root stele cells of 100 and 200 µM Cd-treated plants. In the present study, we also noticed starch in the stroma of plastids treated with Cd. This substance was accumulated in the stele cells treated with 50, 100, and 200 µM CdSO_4_ and also in the cortex cells of 200 µM Cd-treated roots. We hypothesize that starch accumulation may be Cd induced, because starch grains were not observed in the control roots. Interestingly, Barcelo et al. [25] did not observe starch grains in the plastids of six-day-old *Phaseolus vulgaris* root after 15 days of 5 µg/mL CdCl_2_ supplementation. However, similar to our results, Higuchi et al. [47] observed starch accumulation under Cd stress, but in the stem of common reed. Moreover, since the export and allocation of carbon was not disturbed by Cd, these authors suggested that it might be an adaptive response, rather than a result of damage under excess Cd conditions [47,48]. Another feature we observed in the Cd-stressed root cells was oil bodies. They were abundant in the stele of the roots treated with 50 or 100 µM CdSO_4_ and in the cortex of the roots treated with 200 µM CdSO_4_. Increased numbers of osmiophilic globules were also observed in the cytoplasm of cucumber Cd-tolerant cells grown in the continuous presence of Cd [49]. We suggest, that abundance of oil bodies in the Cd-treated cells may be caused by the accumulation of H_2_O_2_ in the root, due to Cd-induced decrease in antioxidative enzyme activity. The possible metabolic link between lipid droplet accumulation, oxidative stress, and alleviation of Cd-induced toxicity was recently described for *Saccharomyces cerevisiae* [50].

In summary, our results showed that Cd treatment had affected the enzyme activity by increasing the PAL activity and lowering of antioxidative enzyme activity and the accumulation of H_2_O_2_ in the root tissues of pea plants. Moreover, increased numbers of structures involved in autophagic cell death (plastolysomes) and oil body formation in the cytoplasm of the stele cells after Cd treatment was observed. In addition, an accumulation of plastid starch granules suggests a possible change of carbohydrate metabolism or carbohydrate distribution in the Cd-treated roots. Furthermore, we observed different distribution of these structures in the 200 µM CdSO_4_ treated root, i.e., accumulation of plastid starch grains and oil bodies in the cortex cells also, increased suberin deposition in the endoderm cell walls, and the relatively lesser amount of oil bodies and plastolysomes in the stele cells of 200 µM Cd-treated roots but also a significantly higher activity of PAL after one day of 200 µM CdSO_4_ treatment. Therefore, we suggest that endodermal cell suberization as well as plastid starch grain and oil body accumulation and PAL activation may be involved in defense reactions against increased concentrations of Cd.

## 4. Materials and Methods

### 4.1. Plant Material and Experimental Design

The seeds of *P. sativum* L. ‘Pegaz’ (Torseed, Poland) were surface sterilized with 75% ethanol for 5 min, followed by 1% sodium hypochlorite for 10 min, washed in water, and sown in wet perlite. Plants were grown in a greenhouse at a monthly average temperature of 24 (±2) °C, watered, when needed, with distilled water and once a week with water solution of 50% Murashige and Skoog [51] medium. The four-week-old plants were supplemented with 10, 50, 100, and 200 µM CdSO_4_ for one week (100 mL of solution per pot—ϕ 15 cm). At the same time, control individuals received equal quantities of distilled water. In each variant of the experiment, at least three pots (each with five plants) were analyzed. The enzyme (CAT, GPX, APX, and PAL) activity was determined after one and seven days of treatment. For microscopic studies the control, 10, 50, 100, and the 200 μM CdSO_4_-treated roots of *P. sativum* after seven days of Cd application were collected. The experiment was carried out in three independent replicates.

### 4.2. Water Content (WC) on a Dry Weight Basis Measurement

Roots of all variants of the experiment were weighed (FW—fresh weight) and then dried at 90 °C in a blow dryer for 24 h. After drying, the samples were re-weighed (DW—dry weight) and water content (WC) of the tissues was determined. WC on a dry weight basis was measured as a ratio between water and the dry mass in tissues. WC was calculated from Formula (1):

(1) $$WC\left(g\times g^{-1}DW\right)=\left(FW-DW\right)÷DW$$

### 4.3. Enzyme Extractions and Assays

All enzyme-extraction steps were carried out at 4 °C. PAL (EC 4.3.1.5) activity was assayed by homogenizing the frozen tissues (0.2 g), with cold mortar and pestle, in a cold solution containing 0.9 mL of 50 mM sodium phosphate buffer (pH 7.0) with 2% (*w*/*v*) polyvinylpolypyrrolidone (PVPP), 2 mM EDTA, and 18 mM β-mercaptoethanol. After centrifugation (15,000× *g* for 20 min at 4 °C), PAL was assayed in the supernatant by measuring the formation of cinnamic acid at 290 nm according to a modified method from Camacho-Cristóbal et al. [52]. PAL activity was defined as 1 µg cinnamic acid per mg protein per 2 h. Control assays did not contain L-phenylalanine.

Extracts of antioxidative enzymes were prepared by homogenizing the frozen roots (0.05 g for GPX; 0.1 g for APX and CAT assays), with cold mortar and pestle, in a cold solution containing 50 mM phosphate buffer (pH 7.0), 1 mM EDTA, 1 mM dithiothreitol (DTT), and 1 mM phenylmethylsulfonyl fluoride (PMSF). In the case of APX assays, 5 mM ascorbate was also added to the extraction solution. After that, the homogenate was centrifuged at 1200× *g* for 20 min at 4 °C and the supernatant was used for the assays. GPX activity was determined by monitoring guaiacol oxidation to tetraguaiacol [53]. The reaction mixture (final volume 3 mL) consisted of 50 mM phosphate buffer (pH 7.0), 0.1 mM H_2_O_2_, 20 mM guaiacol, and 0.025 mL enzyme extract. The reaction was initiated by the addition of hydrogen peroxide, and a monitoring of the increase in absorbance at 470 nm for 3 min was performed (ε = 26.6 mM^−1^cm^−1^). APX activity was determined by monitoring the rate of hydrogen peroxide-dependent oxidation of ascorbic acid [54]. The reaction mixture (final volume 3 mL) consisted of 50 mM phosphate buffer (pH 7.0), 0.1 mM H_2_O_2_, 0.5 mM ascorbate, and 0.15 mL of enzyme extract. The reaction was initiated by the addition of hydrogen peroxide, and monitoring of the decrease in absorbance at 290 nm for 3 min was performed (ε = 13.7 mM^−1^cm^−1^). CAT activity was assayed by monitoring the degradation of hydrogen peroxide [55]. The reaction mixture (final volume 3 mL) consisted of 50 mM phosphate buffer (pH 7.0), 10 mM H_2_O_2_, and 0.2 mL of enzyme extract from root tissue. The reaction was initiated by the addition of enzyme extract, and monitoring of the increase in absorbance at 240 nm for 3 min was performed (ε = 0.36 mM^−1^cm^−1^). To evaluate whether the reaction was enzymatic, a sample extract was boiled and assayed. Protein was assayed according to the method of Bradford [56] using concentrated Bio-Rad micro dye binding reagent (250 μL per 1 mL protein solution). As a standard, bovine serum albumin in concentrations ranging between 2.5 and 30 μg mL^−1^ was used. The absorbance was measured at 595 nm.

### 4.4. Localization of H_2_O_2_ in Pea Roots

We analyzed the primary and lateral roots of pea harvested after seven days of Cd treatment. Localization of H_2_O_2_ was performed by modifying the method of Thordal-Christensen et al. [57]. The fresh, hand-cut samples were placed in 1 mg/mL solution of 3,3′-DAB-HCl, pH 3.8 (Sigma), for 30 min at room temperature. Production of H_2_O_2_ was visualized as a reddish-brown coloration. We conducted tissue fixation of DAB stained tissues in 2.5% glutaraldehyde after rinsing slides for 10 min. Results were analyzed with Nikon microscopy (Eclipse 80i) and a NIS-Elements BR 3.1 program.

### 4.5. Ultrastructural Analysis

Samples of the maturation zone of the lateral root (0, 10, 50, 100, and 200 µM Cd-treated after one week of Cd application; at least one lateral root per plant and at least four plants per treatment) were fixed in 2.5% glutaraldehyde buffered with 0.1 M phosphate-buffered saline (pH 7.4) and post-fixed in 1% osmium tetroxide after rinsing. Dehydrated samples were embedded in Spurr’s resin (Polysciences Inc.). Ultrathin sections were analyzed in a transmission electron microscope (JEM 1400, JEOL) and iTEM 5.1 (Build 2108) with an acceleration voltage of 80 kV. At least four grids with three to four sections for each treatment were observed and photographed. The images that best represented the changes in the ultrastructure of root cells were selected.

### 4.6. Determination of Cd Concentration

After one and seven days of Cd treatment, roots were harvested for Cd-accumulation analysis. Samples were oven-dried and mineralized in a mixture of HNO_3_ and HClO_4_ 3:1 (*v*/*v*) using a temperature step gradient (maximum of 200 °C), for 4–5 h (DK 20, VELP Scientifica). Digests were diluted with deionized water to 25 mL. Cd concentration was measured by a flame atomic absorption iCE 3000 Series spectrometer (Thermo Fisher).

### 4.7. Statistical Analysis

In order to determine differences between groups, an analysis of variance (ANOVA) was performed, followed by the Duncan’s test with the level of significance set at *P* < 0.01. Pearson’s correlation coefficients (R) were estimated between quantitative parameters and Cd concentration used during the experiment. The statistical analysis was carried out using STATISTICA (ver. 13.1 Dell Inc., Tulsa, OK, USA).
